# Excitotoxicity and ALS: New therapy targets an old mechanism

**DOI:** 10.1016/j.xcrm.2024.101423

**Published:** 2024-02-20

**Authors:** Hannah Louise Smith, Helena Chaytow, Thomas Henry Gillingwater

**Affiliations:** 1 Edinburgh Medical School: Biomedical Sciences, University of Edinburgh, Edinburgh, UK; 2 Euan MacDonald Centre for Motor Neuron Disease Research, University of Edinburgh, Edinburgh, UK

## Abstract

Excitotoxicity-induced cell death in motor neurons is a major therapeutic target for amyotrophic lateral sclerosis (ALS). Yan et al.^1^ present a novel compound to specifically disrupt extra-synaptic NMDAR complexes, extending the lifespan of the SOD1^G93A^ ALS mouse and ameliorating cell death.

## Teaser

Excitotoxicity-induced cell death in motor neurons is a major therapeutic target for amyotrophic lateral sclerosis (ALS). Yan et al. present a novel compound to specifically disrupt extra-synaptic NMDAR complexes, extending the lifespan of the SOD1^G93A^ ALS mouse and ameliorating cell death.

## Main text

Amyotrophic lateral sclerosis (ALS) is a highly heterogeneous, fatal motor neuron disease with variable clinical presentation and genetic contributions. Dozens of gene mutations have been linked to ALS, resulting in considerable efforts to develop a toolkit of gene-targeting therapies. However, these treatments are unlikely to be effective for the majority of patients with ALS without a known genetic cause of disease. While numerous molecular pathways are known to be dysregulated in ALS, leading to multiple proposed mechanisms contributing to motor neuron death, there is a disappointing lack of treatment options, even after decades of research.^2^ It seems increasingly likely, therefore, that this heterogeneous and complex disease will require multifactorial approaches to treatment—with combinatorial therapies developed on a personalized-medicine basis. Thus, identifying and targeting molecular pathways relevant across a broad range of patients with ALS, regardless of disease cause, represents an area of much interest. Active progress has been made across the many dysregulated aspects of ALS pathology, from energy metabolism through RNA processing to protein homeostasis and beyond.^3^

**Figure 1:**
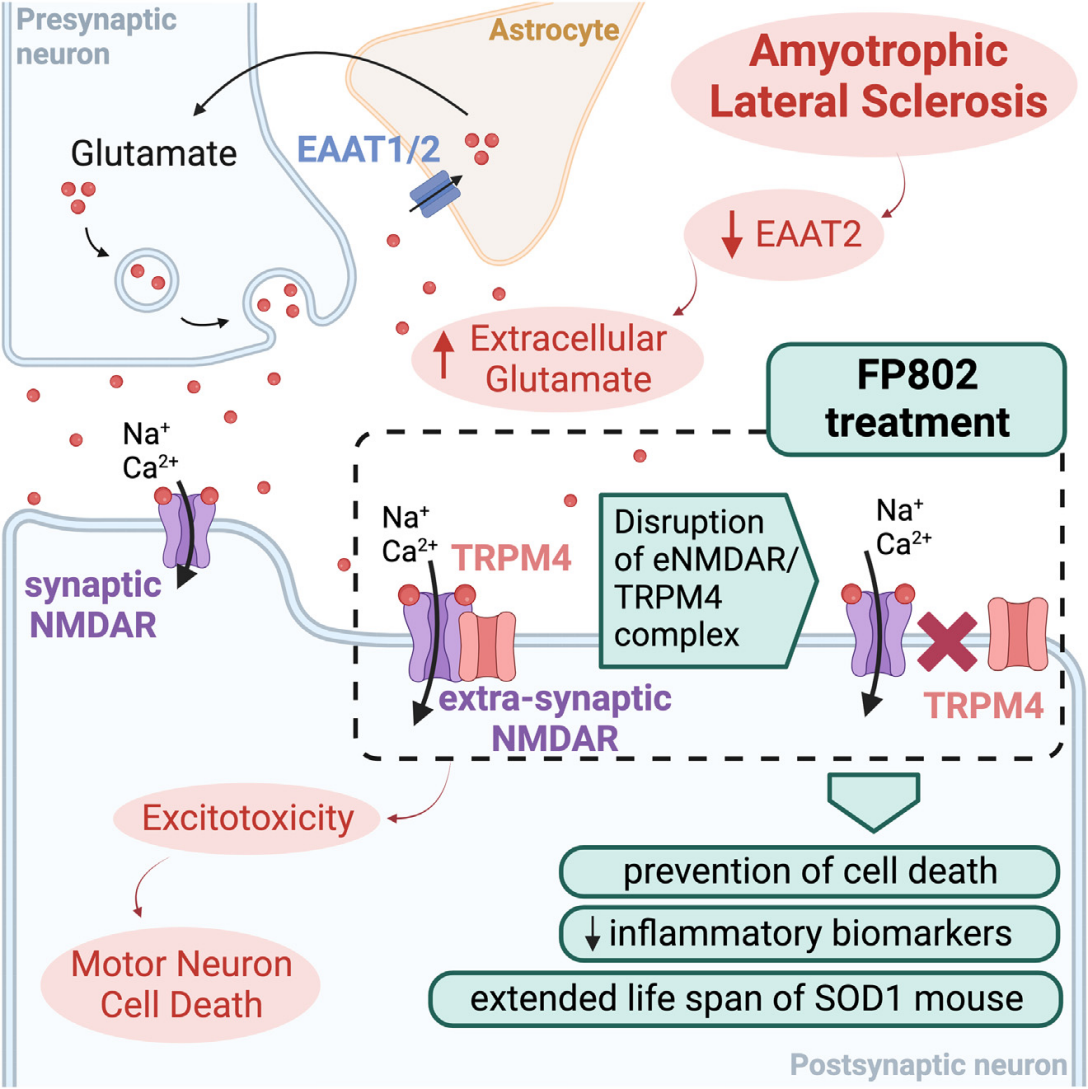
FP802 specifically disrupts the complex between extra-synaptic NMDARs (eNMDARs) and TRPM4, ameliorating ALS model phenotypes by preventing excitotoxicity

Motor neurons in ALS are known to be vulnerable to excessive stimulation by glutamate, which led to the early identification of excitotoxicity as a driver of disease and the development of riluzole. As one of the few licensed treatment options for ALS, riluzole targets glutamate excitotoxicity, preventing motor neuron cell death, albeit with limited improvements in patients’ lifespans.^4^ Glutamatergic stimulation is partly mediated by N-methyl-D-aspartate receptors (NMDARs) in synapses (sNMDARs), but excess glutamate activates cell-death signaling via extra-synaptic NMDARs (eNMDARs), which form complexes with TRPM4.^5^ Targeting eNMDARs to prevent excitotoxicity in neurons has proven challenging to date, largely due to a lack of target specificity, as pharmacological interference with sNMDAR function can be detrimental to normal synaptic function. Previous work by the Bading group demonstrated that a small intercellular domain inside TRPM4, dubbed TwinF, is necessary for the eNMDAR-TRPM4 complex.^5^ Small molecules capable of specifically binding TwinF were computationally derived to disrupt the complex and thus block cell-death signaling in the presence of glutamate. The specificity of these molecules, and their neuroprotective effect from targeting eNMDAR-TRPM4, was previously demonstrated by electrophysiological recording, loss of co-immunoprecipitation of the complex, and reduction of cell death in acute excitotoxic injury models.^5^^,^^6^ In this issue of *Cell Reports Medicine*, Yan et al.^1^ describe FP802, a new TwinF-interfacing compound potentially suitable for clinical use in ALS, with tantalizing pre-clinical findings in mouse and cell models (Figure 1).

Yan et al.^1^ provide evidence that FP802 ameliorates pathology in the SOD1^G93A^ mouse model of ALS. To better mimic conditions in the clinic, treatment was initiated at the onset of motor symptoms. Despite this relatively late time point of therapeutic intervention, there was a significant increase in survival, with improved motor phenotypes over controls, and sparing of motor neurons in the spinal cord. These pre-clinical data demonstrate that targeting eNMDARs can improve key disease outcomes *in vivo*. Furthermore, biomarkers correlating with phenotype progression were ameliorated. Neuroinflammatory markers, increased in the SOD1^G93A^ mouse due to motor neuron death, were reduced in the treatment group. Serum neurofilament light chain, a key biomarker for ALS and a commonly measured parameter in patients with ALS, was also reduced, paving the way for disease monitoring in future clinical trials. The improved pharmacokinetics of FP802 over previous compounds, with good safety profiling and lack of off-target binding, also clearly demonstrate that this drug is a strong candidate for progressing toward clinical trials. However, some questions still remain. Although SOD1-dependent forms of ALS are more vulnerable to excitotoxicity than other forms of the disease, it has long been known that the glutamate reuptake system, particularly GLT-1, is dysregulated across patients with ALS.^7^ It therefore remains to be established whether FP802 treatment has the potential to be equally beneficial in non-SOD1 forms of ALS. In this regard, and also to probe FP802’s applicability for use in humans, Yan et al. generated forebrain organoids from human induced pluripotent stem cells (iPSCs). The organoids derived from patients with ALS, taken from both SOD1-linked and sporadic ALS cases, were over-stimulated with excess NMDA, and both were found to be more susceptible to cell death than healthy controls. Finally, FP802 was neuroprotective against this acute excitotoxic insult, demonstrating initial promise for the compound in a human *in vitro* model alongside the *in vivo* mouse data.

ALS, by its very nature, will likely require combinatorial therapies to meet the wide-ranging challenges presented during disease pathogenesis. Yan et al.^1^ offer a new therapeutic compound, FP802, aimed at a specific aspect of ALS, excitotoxicity, which has exciting potential in such a combinatorial medicine approach. For example, it is possible to envisage a scenario where FP802 could be combined with other emerging therapies to provide better treatment outcomes for patients. Examples of such emerging therapies that might be appropriate for combinatorial use include Tofersen, an antisense oligonucleotide that reduces production of the mutant SOD1 pathological protein in familial SOD1-ALS.^8^ Alternatively, AMX0035 targets mitochondrial and endoplasmic reticulum (ER) stress, slowing progression of symptoms in both sporadic and familial ALS patients.^9^ Similarly, the repurposed drug terazosin increases energy availability to motor neurons, thereby providing neuroprotection.^10^ Given the diverse mechanism of action of these (and other) emerging therapies, a combined approach could conceivably provide a robust disease-modifying treatment option for a wide spectrum of patients with ALS. The identification of FP802 therefore represents a potentially important step toward the future of effective, combinatorial therapies for ALS.
